# Risk of death by suicide following self-harm presentations to healthcare: development and validation of a multivariable clinical prediction rule (OxSATS)

**DOI:** 10.1136/bmjment-2023-300673

**Published:** 2023-05-23

**Authors:** Seena Fazel, Maria D L A Vazquez-Montes, Yasmina Molero, Bo Runeson, Brian M D’Onofrio, Henrik Larsson, Paul Lichtenstein, Jane Walker, Michael Sharpe, Thomas R Fanshawe

**Affiliations:** 1 Psychiatry, University of Oxford, Oxford, UK; 2 Oxford Health NHS Foundation Trust, Oxford, UK; 3 Nuffield Department of Primary Health Care Sciences, University of Oxford, Oxford, UK; 4 Department of Medical Epidemiology and Biostatistics, Karolinska Institute, Stockholm, Sweden; 5 Department of Clinical Neuroscience, Karolinska Institute, Stockholm, Sweden; 6 Stockholm Health Care Services, Stockholm, Sweden; 7 Department of Psychological and Brain Sciences, Indiana University Bloomington, Bloomington, Indiana, USA; 8 School of Medical Sciences, Örebro Universitet, Orebro, Sweden; 9 Psychological Medicine Research Department of Psychiatry, University of Oxford, Oxford, UK

**Keywords:** Suicide & self-harm, Depression & mood disorders, Substance misuse, Adult psychiatry

## Abstract

**Background:**

Assessment of suicide risk in individuals who have self-harmed is common in emergency departments, but is often based on tools developed for other purposes.

**Objective:**

We developed and validated a predictive model for suicide following self-harm.

**Methods:**

We used data from Swedish population-based registers. A cohort of 53 172 individuals aged 10+ years, with healthcare episodes of self-harm, was split into development (37 523 individuals, of whom 391 died from suicide within 12 months) and validation (15 649 individuals, 178 suicides within 12 months) samples. We fitted a multivariable accelerated failure time model for the association between risk factors and time to suicide. The final model contains 11 factors: age, sex, and variables related to substance misuse, mental health and treatment, and history of self-harm. Transparent reporting of a multivariable prediction model for individual prognosis or diagnosis guidelines were followed for the design and reporting of this work.

**Findings:**

An 11-item risk model to predict suicide was developed using sociodemographic and clinical risk factors, and showed good discrimination (c-index 0.77, 95% CI 0.75 to 0.78) and calibration in external validation. For risk of suicide within 12 months, using a 1% cut-off, sensitivity was 82% (75% to 87%) and specificity was 54% (53% to 55%). A web-based risk calculator is available (Oxford Suicide Assessment Tool for Self-harm or OxSATS).

**Conclusions:**

OxSATS accurately predicts 12-month risk of suicide. Further validations and linkage to effective interventions are required to examine clinical utility.

**Clinical implications:**

Using a clinical prediction score may assist clinical decision-making and resource allocation.

**WHAT IS ALREADY KNOWN ON THIS TOPIC**

- Self-harm is associated with a 1-year risk of suicide that is 20 times higher than the general population. Current structured approaches to stratify suicide risk in this population are based on tools developed for other purposes and symptom checklists, and have high false-positive rates. We developed and validated a risk prediction model using population data.

**WHAT THIS STUDY ADDS**

- An 11-item risk model to predict suicide was developed using sociodemographic and clinical risk factors, and showed good discrimination and calibration in external validation. This model was translated into a scalable risk calculator (OxSATS).

**HOW THIS STUDY MIGHT AFFECT RESEARCH, PRACTICE OR POLICY**

- The study underscores the importance of considering probability scores for risk prediction, which are widely used in prognostic tools in cardiovascular and cancer medicine. Future work will need to consider linkage to effective treatments, and whether evidence-based tools can support safety planning, allow for more efficient allocation of clinical resources and act as a screen for more detailed clinical assessment.

## Background

Suicide prevention has focused on combining population-based interventions, including restricting access to means, and targeted approaches focused at high-risk groups.^1^ Among the latter are people who have self-harmed, where the 1-year rate for death by subsequent suicide has been estimated to be at least 20-fold higher than the general population suicide rate.^2^ In a US study, 1.6% of those with clinical self-harm diagnoses went on to die from suicide within the next 12 months and 3.9% died within the next 5 years.^3^

Around 16 million people self-harm annually, so the population impact of preventing future suicide is potentially large.^4^ Current recommended treatments involve resource-intensive specialised psychological therapies, which involve training and multiple sessions.^5^ Most healthcare systems cannot offer gold-standard treatments to all those who self-harm, such as 8–12 sessions of individual psychological treatment. Thus, the assessment of future risk is one approach highlighted to most effectively target resources.^6^ Use of risk assessment tools has been criticised as they demonstrate at best moderate accuracy, can distract clinicians from therapeutic engagement, have high false-positive rates and may miss most suicide deaths if most deaths occur in the low-risk group (‘low risk paradox’). New research has addressed these criticisms.^7^ First, current tools are mostly symptom checklists not developed for suicide risk, and therefore their poor to moderate performance is not indicative of all possible structured risk assessment.^8^ Second, some criticisms are based on mistaken assumptions that these tools replace clinical judgement. In fact, current thinking is that they should complement clinical decision-making, not replace it,^9^ and thereby raise the ceiling of expertise, provide more consistency within and across services, and highlight modifiable risk factors.^10^ In addition, brief tools can act as an initial screen to guide triage decisions.^11^ Third, the lack of evidence on improving outcomes relies on such tools being linked to interventions, and interventions being effective.^10 12^ Fourth, such tools do not invariably have to use risk categories in practice, and can provide probability scores such as risk calculators used in the rest of medicine, including the Framingham risk score for cardiovascular outcomes^13 14^ and prognostic models for cancer survival. This would mitigate against the low risk paradox, which is predicated on dichotomous categories (ie, low vs high) and most suicide deaths being below a categorical threshold. Treatment decisions, however, may continue to rely on binary distinctions, and providing high-quality prediction models allow for guidelines to base these decisions on the best possible evidence.

### Objective

To address limitations in previous research and need for a scalable tool to complement clinical decision-making, we have developed and externally validated a novel risk prediction model for suicide mortality in people presenting to hospital with self-harm.

## Methods

### Study design

We performed a retrospective cohort study linking national Swedish databases, including Patient Register, Cause of Death Register, Longitudinal Integrated Database for Health Insurance and Labour Market Studies, Prescribed Drug Register, National Crime Register and Register of Persons Suspected of Offences.^15^ Eligible individuals were those aged at least 10 years (as it was assumed those under 10 years had their age misclassified), who presented with an emergency visit to hospital or specialist (psychiatric and other) care for non-fatal self-harm (ICD-10 codes X60–X84 and Y10–Y33) between 1 January 2008 and 31 December 2012. For those with more than one such instance within this period, a single index date was selected at random (as this would reflect how any tool would be used in real-world settings). Individuals who died within the hospitalisation period following a self-harm event were excluded as index cases. In addition, for those who self-harmed and then had routine psychiatric follow-up appointments, we used the first self-harm date (as the follow-ups would use the same ICD diagnostic code). We included Y10–Y33 as excluding them has been shown to underestimate rates. However, we excluded people solely categorised as Y34 (where self-harm method was unrecorded) due to its high prevalence in the validation sample (48% vs 19% in the development sample) compared with other self-harm categories and so may have indicated misclassification with respect to the target patient population. Before 1992, the ICD-8/ICD-9 codes E950–959 and E980–987 were also used where necessary (excluding E988–989, the equivalent codes for unrecorded).

### Outcomes

Follow-up information was obtained until 31 December 2013 to ensure at least 1 year of follow-up for all included individuals, up to a maximum of 2 years of follow-up per person. The outcomes were defined as death from suicide within 12 months (primary outcome) and within 6 months of the index date. In common with previous work,^16^ suicide was defined with ICD-10 codes corresponding to death from intentional self-harm (X60–X84) and an event of undetermined intent (Y10–Y33, excluding Y34—unspecified events).

### Risk factors

Potential risk factors comprising sociodemographic information, clinical history and treatment, family psychiatric history and criminal records were obtained from linked national registers (definitions in online supplemental material). We did not use primary care registers, so this information on risk factors was based on hospital or secondary care. Age at the index date was used. Based on previous epidemiological studies, reviews and existing prediction tools for suicide,^3 16–27^ candidate risk factors available in our datasets were allocated into two groups a priori (see online supplemental table 1, including references). One group contained factors to be retained in the model either because a clear association with suicide incidence has been demonstrated in previous systematic reviews^20^ (eg, lifetime self-harm, alcohol use disorder) or to ensure face validity (eg, age, sex). The other group contained the remaining factors, to be selected using backward stepwise selection (5% significance level), where the evidence in previous studies was less certain or inconsistent. All factors were available at the index assessment. We considered models with and without the inclusion of criminal history risk factors, although as criminal history may be more difficult to obtain in other settings, we preferred to exclude these from the final model unless they led to a substantial increase in predictive performance. Correlation between risk factors was not considered as the objective was prediction rather than estimation of individual effects. Sample size considerations included >10 outcomes per predictor for development and >100 outcomes for validation. Blinding for outcomes and predictors was ensured by independently extracting information for each relevant variable.

10.1136/bmjment-2023-300673.supp1 Supplementary data

### Statistical methods

We used a multivariable accelerated failure time model with Weibull errors to investigate associations between risk factors and time to suicide. Given that the proportional hazards assumption was not satisfied for the age variable, this was the most suitable time-to-event model regarding goodness-of-fit. Non-informative censoring at the end of 2-year follow-up was assumed. Risk factors considered as possible predictors were included as covariates in the model without interactions, with the exception of two predefined interaction terms (age×sex and age×lifetime history of self-harm), which were included based on previous research.^2^ Age in years was treated as a continuous variable and included using fractional polynomials to allow for non-linear effects.^28 29^ Backward stepwise selection was used to determine included predictors as described above. We planned multiple imputation for predictors with missing data, but as missing data were negligible, this was not necessary.

We corrected for optimism (overfitting, that is, the tendency of predictive models to perform better in development samples than external populations) by multiplying every estimated model coefficient by a uniform shrinkage factor, calculated as the ratio (model’s χ^2^–df)/model’s χ^2^(30). The fitted model was used to obtain predicted probabilities for all individuals at two prediction horizons: suicide within 6 and 12 months. Internal discrimination assessment was performed using bootstrapping^30^ (subtracting the average difference between discrimination measures from bootstrap-created models evaluated in their bootstrap samples from uncorrected discrimination of the original model) and evaluated using Harrell’s c-statistic.^31^ We also calculated Somers’ D statistic (non-parametric measure of strength and direction of association between ordinal dependent and independent variables) and area under the receiver operating characteristic (ROC) curve. Calibration was assessed by calculating the ratio of observed to expected events (O:E ratio) and by estimating the intercept and slope of a calibration plot between predicted and observed probabilities (split into 20 equal groups) of suicide at 6 and 12 months. For all summary measures, 95% CIs were obtained.

We predefined a cut-off point of ≥5% predicted probability of suicide within 12 months to designate a ‘higher risk’ group. As the majority of individuals’ predicted probabilities fell below this value, we additionally report performance measures (sensitivity, specificity, positive/negative predictive values) using cut-off points of 1% and 2%, to aid interpretation. The latter were preferred after taking into account suicide incidence, and improving sensitivity. We created a web-based risk calculator to allow easy calculation of predicted probabilities. Data were extracted using SAS software and statistical analysis carried out using StataSE V.16 (StataCorp).

### Validation

A geographical validation sample was obtained using the residential location of the individual at the time of episode (or the year before or after if missing).^32^ This was preferred to a completely random split sampling (at the individual level) for development and validation, which has been shown to overestimate model performance, partly as the distribution of risk factors and outcome prevalence will inevitably be similar and partly because model development will be in a smaller dataset, increasing risk of overfitting.^33^ Four non-overlapping groups of geographical regions were defined according to population density. The regions were based on the counties of Sweden and derived from the first two digits of the SAMS code, except for Malmo, Gothenburg, North and South Stockholm City for which four SAMS digits were used. Online supplemental table 2 gives details of the four groups created: (1) major urban centres, (2) counties with major urban centres removed, (3) counties with small population density and (4) counties with medium population density. One region was randomly selected from each of the first three groups and then sequentially from group 4, until the number of suicides within 6 months since index reached 100 or more events.^9 34^ This defined an ‘external validation sample’; data from the remaining regions were used for model development. Predictions in the validation sample were calculated using the equation of the development model sample. The same discrimination and calibration measures as described above were calculated in external validation.

Transparent reporting of a multivariable prediction model for individual prognosis or diagnosis (TRIPOD) guidelines were followed for the design and reporting of this work.^35^

## Findings

The cohort consisted of 53 172 individuals, of whom 37 523 were assigned to the model development sample and 15 649 to external validation. In model development, 267 (0.7%) died from suicide within 6 months, 391 (1.0%) within 12 months of the index date and 540 (1.4%) in the 2-year follow-up period. In external validation, corresponding figures were 108 (0.7%), 178 (1.1%) and 251 (1.6%), respectively (see online supplemental table 2 for suicide prevalence at each prediction horizon within the regions comprising the development and validation samples). Median age in development and external validation samples was 32.2 and 32.5 years, respectively, and the proportion of females was 55% and 56%, respectively. Forty-four per cent of the development and 47% of the validation samples had a mental health diagnosis in the previous 12 months (table 1).

**Table 1:** Baseline characteristics of the study population

General demographics	Development sample N=37 523		Validation sample N=15 649	
	N	%	N	%
Age in years, median (IQR)*	32.2	21.4–48.9	32.5	21.4–49.1
Individuals under 16 years	2224	5.9	864	5.5
Sex, female*	20 561	54.8	8685	55.5
**Substance misuse**				
Current or lifetime alcohol use disorder, excluding alcohol intoxication*	7257	19.3	3025	19.3
Current or lifetime drug use disorder (including drug intoxication)*	8384	22.3	3672	23.5
Alcohol intoxication at index	1416	3.8	815	5.2
**Living status†**				
Living with other adults	14 151	37.7	5697	36.4
Living with children	15 493	41.3	6494	41.5
**Treatment in the past 3 months**				
Any psychotropic medication	20 888	55.7	9537	60.9
Antidepressant treatment	12 527	33.4	6009	38.4
Antipsychotic treatment	4259	11.4	1834	11.7
Mood-stabiliser treatment	677	1.8	310	2.0
**Physical health problems**				
New cancer diagnosis	411	1.1	149	1.0
**History of self-harm**				
*Method of index self-harm event*‡				
Any psychotropic medication overdose	1360	3.6	539	3.4
Cutting	4925	13.2	1614	10.3
Hanging, strangulation or suffocation	308	0.8	150	1.0
Drowning	51	0.1	23	0.2
Lifetime history of self-harm prior to index*	11 277	30.1	5372	34.3
History of self-harm in the past 12 months prior to index*	4281	11.4	1994	12.7
*Number of lifetime prior self-harm episodes, including the index self-harm event*				
1–2 episodes	31 740	84.6	12 879	82.3
3+ episodes	5783	15.4	2770	17.7
Overnight admission	16 991	45.3	7531	48.1
Time between episodes ≤1 month	1929	5.1	838	5.4
**Mental health in the past 12 months**				
Any psychiatric disorder (except substance use disorders)	16 472	43.9	7281	46.5
Serious psychiatric disorder	3006	8.0	1305	8.3
**Criminal/violence/legal issues**				
Lifetime criminal record for any crime	13 451	35.9	5974	38.2
Criminal record for any crime in past 12 months	3816	10.2	1809	11.6
Lifetime arrest history for any crime	15 068	40.2	6506	41.6
Arrest history for any crime in past 12 months	6416	17.1	2818	18.0
Lifetime criminal record for violent crime	5729	15.3	2576	16.5
Criminal record for violent crime in past 12 months	897	2.4	416	2.7
Lifetime arrest history for violent crime	5402	14.4	2266	14.5
Arrest history for violent crime in the past 12 months	2141	5.7	872	5.6
**Family history**				
Family history of suicide	1118	3.0	530	3.4
Family history of any psychiatric disorder	15 112	40.3	6503	41.6

Values are all numbers and percentages, except for age for which median years and IQR are reported. Criminal arrest refers to being charged for an offence.

*Core factor, kept in the final model independently of its statistical significance or predictive strength.

†Living status information was missing for 641 (2%) individuals in the development sample and 199 (1%) in the validation sample. Individuals under 16 years old with missing living status were reclassified as ‘living with other adults’ (2049 (92%) of the under 16s in the development sample; 815 (94%) in the validation sample). Of those with missing living status, two individuals died from suicide more than 12 months since index in the development sample, zero in the validation sample. For variables psychiatric treatment, mental health, family history, new cancer diagnosis and self-harm method, when there was no information, it was assumed to indicate the variable did not occur rather than a missing value.

‡It was possible for an individual’s index self-harm event to be coded under more than one of the methods listed. The majority of individuals not included in any of the listed categories had either non-psychotropic medicine overdose or an unspecified method of index self-harm. Drowning was too rare to enter into the multivariable model.

As method of index self-harm, cutting was used by 13% of the development sample and 10% of the validation sample.

Among those who died from suicide, the median survival time following the index date was 11 months (IQR 3–24). During follow-up, 1135 (3%) individuals died of other causes, with a median survival time of 9 months (IQR 4–15). Similarly, in the validation sample, the median survival time was 11 months (IQR 3–24) among those who died from suicide and 9 months (IQR 3–16) among the 494 (3%) individuals who died of other causes.

The fitted model contained 11 risk factors (table 2). Factors associated with higher risk of suicide included male sex, current or lifetime drug use disorder, recent psychiatric disorder, recent psychotropic medication, lifetime and history of self-harm, overnight admission, and an index self-harm method attributed to psychotropic medication overdose and to hanging, strangulation or suffocation. The association with age was non-linear, with low risk at the youngest ages but a sharp increase in risk post-adolescence (online supplemental figure 1). No interaction terms were retained. The full model prediction equation is presented in online supplemental table 3. Unadjusted associations between candidate predictors and outcome are shown in online supplemental table 4. Our preferred final model did not include criminal history variables, as their effect on performance measures was small (online supplemental tables 5 and 6).

**Table 2:** Risk factors (and their HRs and 95% CIs) in the final multivariable model to predict risk of death by suicide after an emergency treatment for self-harm

	Adjusted HR	95% CI		P value
General demographics				
(Age at index/10)^−2^*	0.015	0.005	0.046	<0.001
Female*	0.50	0.42	0.59	<0.001
Substance misuse				
Current or lifetime alcohol use disorder (excluding alcohol intoxication)*	0.97	0.80	1.18	0.785
Current or lifetime drug use disorder (including drug intoxication)*	1.36	1.13	1.64	0.001
Treatment in the past 3 months				
Any psychotropic medication	2.14	1.68	2.74	<0.001
History of self-harm				
Overdose of any psychotropic medication as part of index self-harm episode	1.52	1.02	2.26	0.039
Hanging, strangulation or suffocation as part of index self-harm episode	2.64	1.42	4.90	0.002
Lifetime history of self-harm prior to index*	1.16	0.94	1.43	0.172
History of self-harm in the past 12 months prior to index*	1.36	1.07	1.73	0.013
Overnight admission	1.76	1.47	2.11	<0.001
Mental health in the past 12 months				
Any psychiatric disorder except substance use disorders	1.66	1.37	2.01	<0.001

*Core factor.

### Internal performance

After correcting for optimism, which is the tendency of predictive models to perform better in development samples than external populations, Harrell’s c-statistic for the overall internal discrimination performance was 0.76 (95% CI 0.73, 0.78). That is, in two randomly selected individuals, one with the outcome and one without, 76% of the time the model will estimate a higher risk for someone who will die by suicide in 12 months than for the person who will not. Somers’ D statistic was 0.52 (0.47, 0.56). Online supplemental table 6 shows further information on these performance measures before and after adjusting for optimism. Online supplemental figure 2 presents the ROC curves and corresponding area under the curve (AUC) when using the model to predict risk of suicide at 6 and 12 months in the development sample. For both prediction horizons, the predicted risk across the whole cohort was generally much smaller than the prespecified cut-off point of 5% (median predicted risk within 6 months 0.5% (IQR 0.2%–1.1%); within 12 months, 0.8% (IQR 0.4–1.6%)). Of the two alternative cut-off points (1% and 2%), 1% was the optimal option. Online supplemental figure 2 illustrates the sensitivity and specificity of these three cut-off points for the development sample and online supplemental table 7 presents the classification measures for all combinations of cut-off point and prediction horizon.

The model showed good internal calibration (see online supplemental figure 3 for calibration plots, intercepts, slopes and O:E ratios in development sample).

### External performance

**Figure 1:**
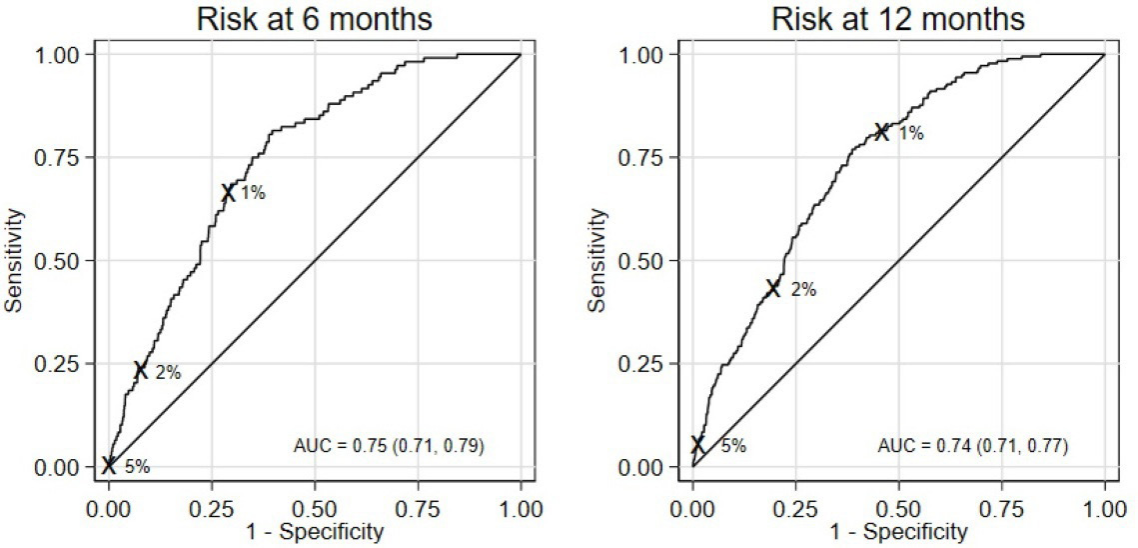
ROC curve and AUC (95% CI) for the final model evaluated in the external validation sample at 6 and 12 months. Sensitivity and specificity for the risk thresholds considered (1%, 2%, 5%) are shown. AUC, area under the curve; ROC, receiver operating characteristic.

The predictive performance of the final model in external validation was good. Harrell’s c-statistics for 6-month and 12-month prediction horizons were 0.77 (0.74, 0.79) and 0.77 (0.75, 0.78), respectively. Corresponding Somers’ D statistics were 0.53 (0.48, 0.58) and 0.53 (0.49, 0.57). Figure 1 presents the ROC curves and corresponding AUCs when using the model to predict risk of suicide at 6 and 12 months (which were very similar to Harrell’s c-statistics, as expected). Sensitivity and specificity for cut-off points 1, 2 and 5% are also indicated in figure 1. In general, classification measures in external validation were similar to those in the development sample. For the preferred cut-off point of 1%, the model’s sensitivity and specificity to predict risk of suicide at 6 months were 68% (58%, 76%) and 71% (70%, 72%), respectively; at 12 months, 82% (75%, 87%) and 54% (53%, 55%), respectively (see online supplemental table 8 for other classification measures, including positive/negative predictive values).

**Figure 2:**
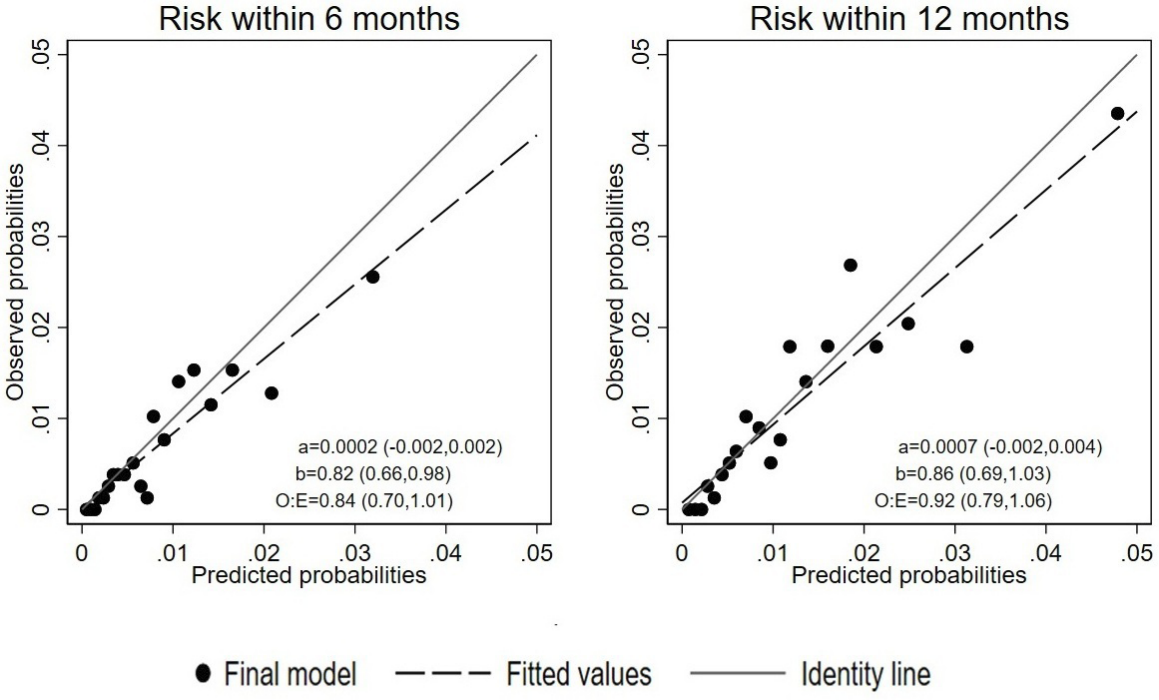
Calibration plots for the final model’s external validation. a/b, calibration intercept and slope (95% CI); O:E, ratio of observed to expected events (95% CI).

Figure 2 presents the calibration plots for risk prediction within 6 and 12 months together with the corresponding intercepts, slopes and O:E ratios. Although external calibration was adequate, there was some indication of overprediction at higher predicted probabilities at the 6-month time point (O:E 0.84 (0.70, 1.01); calibration slope 0.82 (0.66, 0.98)). The model was better calibrated for predicting suicide in the longer term (O:E 0.92 (0.79, 1.06); calibration slope 0.86 (0.69, 1.03)).

## Discussion

In a sample of 53 172 individuals who self-harmed, we developed and externally validated a risk prediction model for death by suicide in the 6 and 12 months after self-harm. Within the 2 years following a self-harm episode, we found that around 1 in 70 people died from suicide, with around 1 in 100 in the 12 months following the self-harm episode. We reported risk factors for suicide in those who self-harmed, and the final model included 11 predictors. These included age and sex, and five relating to recent suicidal behaviours. The performance of the model in external validation was good, with c-index at 6 and 12 months of 0.77. Based on a cut-off of 1%, at 6 months, sensitivity was 68% and specificity was 71%.

There are other tools aimed at the self-harm population, but they are not widely implemented as validation information is lacking, and they have relatively poor performance. The ReACT^26^ tool scored 83% of the derivation sample and 73% of the external sample of repeat self-harm patients as elevated risk, so it lacks discrimination and has high false-positive rates (ie, 1−specificity). In contrast, our model with a 1% cut-off and a 6-month prediction horizon has a false-positive rate of 29%. Another tool, the Manchester Self Harm Rule,^18^ is based on four items, one of which is an overdose with benzodiazepines, and an individual scores as high risk if positive on one item. Benzodiazepines are now less widely prescribed than when data to develop this tool were collected (1997–2001).^36^ The Suicide Assessment Scale^27^ was developed to detect changes in suicidal behaviour based on 20 symptoms grouped in five categories (affect, bodily states, control and coping, emotional reactivity, and suicidal thoughts and behaviour). These variables are not readily available in a clinical setting. The scale was developed and assessed on small samples.^37^

Strengths of the current model include its large sample size. In the development, we investigated 37 523 persons who self-harmed and 391 suicides within 12 months. In external validation, we examined 178 suicides within 12 months. Further, we implemented some methods which are novel in suicide prediction research. These include prespecification of the predictors and the analytical approach, avoiding univariable modelling for selection of predictors and instead combining backward selection and shrinkage techniques, reporting a full range of discrimination and calibration measures, and testing the incremental predictive performance of candidate predictors. Age was included in the model using a non-linear approach, which is also novel and reflected the effect of age more precisely than previous models. Many of the included predictors were prevalent in less than one in five persons who self-harmed, including methods of index self-harm, history of self-harm in the previous 12 months, and diagnosed alcohol use disorder, which would argue against some criticisms of these tools that they rely on common risk factors, and hence lack clinical utility. Future work could consider comparison with other models; external validation and recalibration of other models were not within the scope of this study.

Without linkage to interventions, implementing a risk prediction model on its own will not improve outcomes.^10^ Future work will need to consider how the tool can be used, at what point, and how it can be linked to treatment. One approach recommended is that such models act as screens for more detailed assessment of risks and needs (partly because healthcare systems do not have the resources to conduct this on all persons presenting with self-harm or suicidal ideas^7^). Furthermore, such models are intended to support clinical decision-making rather than undermining the therapeutic relationship by taking a checklist approach. Psychosocial needs should be part of any assessment^5^ and safety planning is central to management. Even then, for any tool to improve outcomes, interventions will need to be effective and scalable.

From a clinical perspective, one strength of a prediction model is that it can improve consistency, especially in busy clinical settings and where assessment is conducted by people with different professional and training backgrounds, anchor decisions in empirical evidence, highlight the role of certain modifiable factors, and provide an opportunity to transparently discuss risk with patients and their carers. In addition, it may improve assessment as clinicians have been shown to be too optimistic about risk^38 39^ and place excess weight on recent factors. We have translated the model into an online risk calculator, Oxford Suicide Assessment Tool after Self-harm (OxSATS; https://oxrisk.com/oxsats/) for further research. This is freely available, incorporates the 11 risk factors in the model using calendar age and mostly binary items, and provides probability scores for 6 and 12 months of risk of suicide death after a healthcare-presenting self-harm episode.

If categories are used, the prevention paradox does not apply to this model. Of those who died from suicide, 32% would have been classified at low risk at 6 months and 18% at 12 months assuming a 1% cut-off. This is a considerable advance from other models where >50% are typically in the low-risk category. At the same time, it underscores the importance of using probability scores for risk assessment, which provide another way of considering risk clinically that is widely used in cardiovascular and cancer medicine.

Limitations include that some risk factors for suicide after self-harm, including psychological symptoms (eg, hopelessness) and assessment questions about future risk,^20^ are not included as these are difficult to ascertain retrospectively in order to develop models with adequate statistical power. Although these factors are associated with suicide risk, it is not certain to what extent they are independent of other predictors. Another problem with these factors is that their reliability may not be high, and they will lead to a longer and more complex tool even if their incremental validity is demonstrated. Another consideration is the positive predictive value of this tool was low if categories are used, which is expected as the suicide base rate is low. Thus, the consequences of an elevated categorical score should not be harmful as they will lead to unnecessary interventions for many persons, and not simplistically used to determine admission. Low positive predictive values underscore our recommendation to consider the probability score, which is not influenced by cut-offs. A final important limitation is model generalisability, and outside of Sweden. This is an empirical question, but we note that the prevalence of key predictors (eg, lifetime self-harm of 31% in the whole cohort) and their links with suicide are associated with low to moderate between-study heterogeneity in a meta-analysis.^20^ Different pathways to care and risk factor distributions, however, may influence how much this model shrinks in new populations. Local studies are recommended to test performance and consider recalibration as part of an implementation strategy, especially since there was some minor overprediction in the external validation sample. Competing risks (eg, all-cause death) were not considered but it is unlikely they could have an effect on our findings due to the short time frames considered and the median age of 32.3 years in the whole cohort. Finally, decision curve analysis should inform the risk probability threshold in future work.

In conclusion, we have developed and externally validated a scalable tool to predict suicide after self-harm presentations with good measures of discrimination and calibration. The tool is based on 11 predictors, and has been translated into a simple online tool with probability scores for suicide at 6 and 12 months after a self-harm presentation.

## Clinical implications

Using a clinical prediction model may assist clinical decision-making and resource allocation. Probability scores, if calibration has been tested and shown to be good, provide one way to stratify risk in a clinically feasible way and underscore safety planning.

## Data Availability

Data may be obtained from a third party and are not publicly available. Data may be obtained from a third party and are not publicly available. The Public Access to Information and Secrecy Act in Sweden prohibits us from making individual-level data publicly available. Researchers who are interested in replicating our work can apply for individual-level data from: Statistics Sweden (mikrodata@scb.se) for data from the Total Population Register; the Swedish National Council for Crime Prevention (statistik@bra.se) for data from the Register of People Suspected of Offences; the Swedish Prison and Probation Service (hk.fou@kriminalvarden.se) for data from the Prison and Probation Register; and the National Board of Health and Welfare (registerservice@socialstyrelsen.se) for data from the Patient Register, the Prescribed Drug Register and the Cause of Death Register.
