# Supplementary material for: Risk of death by suicide following self-harm presentations to healthcare: development and validation of a multivariable clinical prediction rule (OxSATS)
Source: BMJ Ment Health. 2023 May 23;26(1):e300673. doi: 10.1136/bmjment-2023-300673 (PMC10335583; doi:10.1136/bmjment-2023-300673)
Supplement: Supplementary data [file bmjment-2023-300673supp001.pdf]

Supplementary Table 1: Variable definitions

**Outcome definition**	
Death from suicide	Death within 12 months and within 6 months of index self-harm event; ICD-10: X60-X84 (intentional self-harm) or Y10-Y33 (events of undetermined intent)
**General demographics**	
Age*	In years, at time of index date; individuals less than 10 years excluded
Sex*	Coded as male or female
**Substance misuse**	
Current or lifetime alcohol use disorder, excluding acute intoxication*	Any of the following ICD codes at any time at or before index date (acute intoxication (F10.0) excluded)<br>ICD-10 codes: F10.1-F10.6<br>ICD-9: 291, 303.9, 305.0 (nondependent alcohol<br>ICD-8: 291, 303.0, 303.1, 303.2, 303.9
Current or lifetime drug use disorder*	Any of the following ICD codes, at any time at or before index date<br>ICD-10 codes: F11-F16, F18-19 (subdivisions .1-.7)<br>ICD-9: 292, 304, 305.2-305.9 (nondependent drug use)<br>ICD-8: 304
Alcohol intoxication at index	At time of index date, ICD-10: F10.0
**Living status**	Obtained from LISA register, which classifies adults as 16 and older
Living with adult	Living with at least one other individual aged 16+ years. All individuals under 16 years old with missing living status were reclassified as 'living with adult'
Living with children	Living with at least one individual under 16 years old
**Treatment in the past three months**	Dispensed in the three months prior to index date
Any psychotropic medication	Any prescription of antidepressant, antipsychotic or mood stabiliser medication (defined as below)
Antidepressant treatment	ATC: N06A
Antipsychotic treatment	ATC: ATC: N05A, excluding N05AN01, N05AH02
Mood stabilizer treatment	ATC: N03AG01, N03AX09, N03AF01, N03AN01
**Physical health problems**	

New cancer diagnosis	An instance of a cancer diagnosis (ICD-10: C00-D48) occurring within 12 months before index date that is not a relapse (recoding) of a previous diagnosis made in the preceding 2 years
**History of self-harm**	
Any psychotropic medication overdose	Psychotropic medication overdose (ICD-10: X61, Y11) as the method of harm of the index event
Cutting	Cutting (i.e. sharp object, ICD-10: X78 or Y28) as the method of harm of the index event
Hanging, strangulation or suffocation	Hanging, strangulation or suffocation (ICD-10: X70 or Y20) as the method of harm of the index event
Drowning	Drowning or submersion (ICD-10: X71 or Y21) as the method of harm of the index event
Lifetime history of self-harm prior to index*	Any instance of self-harm occurring prior to the index date<br>ICD-10 codes: X60-X84, Y10-Y33<br>ICD-9 codes: E950-E959 and E980-E987<br>ICD-8 codes: E950-E959 and E980-E987
History of self-harm in the past 12 months prior to index*	Any instance of self-harm (ICD-10 codes: X60-X84, Y10-Y33) occurring within the 12 months before the index date
Number of lifetime prior self-harm episodes	1-2 episodes vs 3+
Overnight admission	Binary indicator of whether the index event resulted in an overnight hospital admission
Time between episodes <= 1 month	Any instance of self-harm (ICD-10 codes: X60-X84, Y10-Y33) occurring within one month before the index date (for those with an additional self-harm episode, prior to the index date)
**Mental health in the past 12 months**	
Any psychiatric disorder except substance use disorders	ICD-10: F00-F09, F20-F99 (Mental and behavioural disorders, excluding those due to psychoactive substance use)
Serious psychiatric disorder	ICD-10: F20-F29 (Schizophrenia, schizotypal and delusional disorders), F30-F31 (Mood [affective] disorders)
**Criminal/violence/legal issues**	Obtained from National Crime Register; individuals younger than 15 (the age of criminal responsibility) are excluded from the definitions below
Lifetime criminal record for any crime	Conviction for any crime before the index date
Criminal record for any crime in past 12 months	Conviction for any crime within 12 months before the index date

Lifetime arrest history for any crime	Arrest for any crime before the index date
Arrest history for any crime in past 12 months	Arrest for any crime within 12 months before the index date
Lifetime criminal record for violent crime	Conviction for any of the following before the index date: attempted/completed/aggravated forms of homicide, manslaughter, unlawful threats, harassment, robbery, arson, assault, assault on an official, kidnapping, stalking, coercion, all sexual offences
Criminal record for violent crime in past 12 months	Conviction for any violent crime, as listed above, within 12 months before the index date
Lifetime arrest history for violent crime	Arrest for any violent crime, as listed above before the index date
Arrest history for violent crime in the past 12 months	Arrest for any violent crime, as listed above, within 12 months before the index date
**Family history**	
Family history of suicide	Death from suicide (ICD-10: X60-X84, Y10-Y33) of any parent, sibling or child before the index date
Family history of any psychiatric disorder	Diagnosis of psychiatric disorder (ICD-10 F00-09, F20-F99) of any parent, sibling or child before the index date

Note: all variables except age were coded as binary variables. Those marked with an asterisk (*) are core risk factors that were included in the final model irrespective of statistical significance.

ATC: Anatomical Therapeutic Chemical Classification System

ICD: International Statistical Classification of Diseases and Related Health Problems

LISA: Longitudinal integrated database for health insurance and labour market studies, https://www.scb.se/lisa-en

Supplementary Table 2: Deaths by suicide within the regions forming the development and validation samples (prevalence)

Group 1	Group 2	Group 3	Group 4
Major urban centres	Counties with major urban centres removed	Counties with small population	Counties with medium population
Development sample			
Gothenburg<br>[18, 0.7%] {24, 0.9%}*	Stockholm County Other<br>[51, 0.7%] {88, 1.2%}	Kronoberg<br>[13, 1.5%] {15, 1.7%}	Uppsala<br>[12, 0.6%] {15, 0.7%}
Stockholm City North<br>[13, 0.7%] {21, 1.1%}	Skåne Other<br>[37, 0.8%] {55, 1.1%}	Gotland<br>[2, 0.5%] {2, 0.5%}	Södermanland<br>[6, 0.3%] {10, 0.5%}
Stockholm City South<br>[24, 0.8%] {32, 1.1%}		Blekinge<br>[8, 0.9%] {9, 1.1%}	Jönköping<br>[11, 0.6%] {17, 0.9%}
			Kalmar<br>[17, 1.3%] {24, 1.9%}
			Örebro<br>[11, 0.8%] {15, 1.1%}
			Dalarna<br>[12, 0.9%] {16, 1.2%}
			Gävleborg<br>[7, 0.5%] {14, 1.0%}
			Västernorrland<br>[8, 0.8%] {11, 1.1%}
			Västerbotten<br>[9, 0.7%] {10, 0.8%}
			Norrbotten<br>[8, 0.5%] {13, 0.8%}
External validation sample			
Malmö<br>[9, 0.6%] {25, 1.7%}	Västra Götaland Other<br>[39, 0.7%] {50, 0.9%}	Jämtland<br>[4, 0.8%] {6, 1.2%}	Östergötland<br>[14, 0.5%] {24, 0.9%}
			Halland<br>[7, 0.5%] {11, 0.7%}
			Värmland<br>[18, 1.1%] {33, 2.0%}
			Västmanland<br>[17, 0.8%] {29, 1.4%}

* [Within 6 months] {Within 12 months}

Supplementary Figure 1: Non-linear effect of age, shown for the hazard function

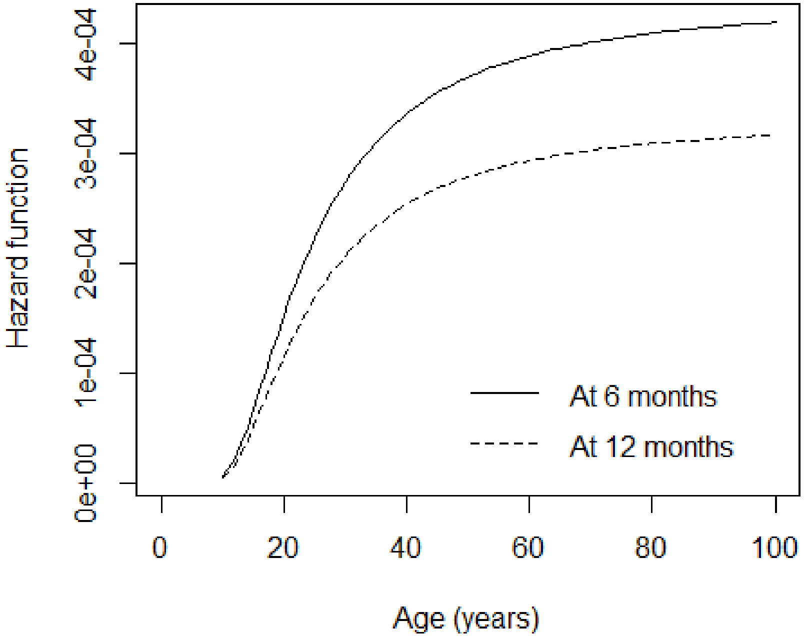

Supplementary Table 3: Parameters (and their 95% confidence intervals) to calculate predicted risk of death by suicide within 6 and 12 months after an emergency treatment for self-harm

	**Parameter (β)**	**95% confidence interval**	
**General demographics**			
(Age at index/10)$^{-2}$*	-4.20	-5.32	-3.08
Sex, female*	-0.70	-0.87	-0.53
**Substance misuse**			
Current or lifetime alcohol use disorder (excluding alcohol intoxication)*	-0.03	-0.22	0.17
Current or lifetime drug use disorder (including drug intoxication)*	0.31	0.12	0.50
**Treatment in the past three months**			
Any psychotropic medication	0.76	0.52	1.01
**History of self-harm**			
Any psychotropic medication overdose	0.42	0.02	0.82
Hanging, strangulation or suffocation	0.97	0.35	1.59
Lifetime history of self-harm prior to index*	0.15	-0.06	0.36
History of self-harm in the past 12 months prior to index, 1+ vs none*	0.31	0.07	0.55
Overnight admission	0.57	0.38	0.75
**Mental health in the past 12 months**			
Any psychiatric disorder except substance use disorders	0.51	0.31	0.70
**Other parameters in the model**			
Intercept	-6.49	-6.79	-6.20
Shape parameter	0.60	0.55	0.65

*Core factors

The formula to calculate the risk of death by suicide within t months after an emergency treatment for self-harm is: $\Pr(suicide\ within\ t\ months) = 1 - \exp(-\exp(linpred_{sui})t^{0.595})$

where $linpred_{sui} = -6.49 + \sum \beta * Risk\ Factor$ and $t = 6$ or 12.

The baseline survivor function is given by $S_0(t) = \exp(-0.0015 * t^{0.595})$. At 6 and 12 months this is $S_0(6) = 0.996$ and $S_0(12) = 0.993$, respectively.

Binary risk factors are coded as 1 if present and 0 if absent.

Supplementary Table 4: Unadjusted associations between candidate predictors and outcome in the development sample

	Hazard Ratio	95% CI for the hazard ratio		p-value	Weibull shape parameter	95% CI for the Weibull shape parameter	
**General demographics**							
Age (years)[1]	1.02	1.02	1.03	<0.001	0.5948	0.5472	0.6465
Female[1]	0.55	0.46	0.65	<0.001	0.5909	0.5435	0.6424
**Substance misuse**							
Current or lifetime alcohol use disorder, excluding alcohol intoxication[1]	2.04	1.70	2.44	<0.001	0.5906	0.5432	0.6421
Current or lifetime drug use disorder (including drug intoxication)[1]	2.36	1.99	2.81	<0.001	0.5908	0.5435	0.6424
Alcohol intoxication at index	0.77	0.47	1.26	0.298	0.5901	0.5428	0.6416
**Living status**							
Living with other adult	0.64	0.53	0.77	<0.001	0.5898	0.5424	0.6412
Living with children	0.55	0.46	0.66	<0.001	0.5903	0.5429	0.6418
**Treatment in the past three months**							
Any psychotropic medication	3.97	3.17	4.97	<0.001	0.5923	0.5448	0.6439
Antidepressant treatment	2.17	1.83	2.57	<0.001	0.5907	0.5433	0.6422
Antipsychotic treatment	2.30	1.88	2.81	<0.001	0.5908	0.5434	0.6423
Mood stabilizer treatment	2.10	1.35	3.29	0.001	0.5901	0.5428	0.6416
**Physical health problems**							
New cancer diagnosis	1.18	0.56	2.49	0.664	0.5901	0.5427	0.6415
**History of self-harm**							
Method of self-harm at index							
Any psychotropic medication overdose	1.33	0.90	1.98	0.153	0.5900	0.5427	0.6415
Cutting as a method of harm	0.54	0.39	0.74	<0.001	0.5901	0.5428	0.6416
Hanging, strangulation or suffocation	2.30	1.23	4.30	0.009	0.5901	0.5428	0.6416
Lifetime history of self-harm prior to index[1]	2.15	1.82	2.55	<0.001	0.5901	0.5427	0.6415
History of self-harm in the past 12 months prior to index[1]	2.21	1.81	2.71	<0.001	0.5902	0.5429	0.6417

Number of lifetime prior self-harm episodes, >1 vs exactly 1	2.17	1.80	2.61	<0.001	0.5904	0.5430	0.6419
Overnight admission	2.26	1.89	2.69	<0.001	0.5903	0.5430	0.6418
Time between lifetime episodes, <=1 vs >1 month	1.99	1.50	2.65	<0.001	0.5909	0.5436	0.6424
**Mental health in the past 12 months**							
Any psychiatric disorder except substance use disorders	2.53	2.12	3.03	<0.001	0.5903	0.5430	0.6418
Serious psychiatric disorder	1.81	1.42	2.32	<0.001	0.5907	0.5433	0.6422
**Criminal/violence/legal issues**							
Lifetime criminal record for any crime	2.01	1.70	2.38	<0.001	0.5903	0.5429	0.6418
Criminal record for any crime in past 12 months	1.48	1.16	1.88	0.002	0.5904	0.5431	0.6419
Lifetime arrest history for any crime	1.64	1.39	1.95	<0.001	0.5901	0.5428	0.6416
Arrest history for any crime in past 12 months	1.49	1.22	1.82	<0.001	0.5902	0.5428	0.6416
Lifetime criminal record for violent crime	1.43	1.16	1.76	0.001	0.5901	0.5428	0.6416
Criminal record for violent crime in past 12 months	1.16	0.70	1.94	0.564	0.5902	0.5428	0.6417
Lifetime arrest history for violent crime	1.52	1.23	1.87	<0.001	0.5901	0.5427	0.6415
Arrest history for violent crime in the past 12 months	1.62	1.21	2.18	0.001	0.5903	0.5430	0.6418
**Family history**							
Family history of suicide	1.52	1.01	2.29	0.045	0.5902	0.5428	0.6416
Family history of any psychiatric disorder	1.06	0.89	1.25	0.538	0.5901	0.5428	0.6416

[1]Core factor, kept in the final model independently of its statistical significance or predictive strength; Univariate association between drowning as a method of index self-harm and suicide was not assessed because none of the individual who died by suicide use drowning at index.

Supplementary Table 5: Risk factors (and their hazard ratios and 95% confidence intervals) in the final multivariate model to predict risk of death by suicide after an emergency treatment for self-harm, when criminal history variables are considered (Crime model).

	**Hazard Ratio**	**95% confidence interval**		**p-value**
**General demographics**				
(Age at index/10)$^{-2}$*	0.016	0.005	0.049	<0.001
Sex, Female*	0.51	0.42	0.61	<0.001
**Substance misuse**				
Current or lifetime alcohol use disorder (excluding alcohol intoxication)*	0.95	0.78	1.15	0.578
Current or lifetime drug use disorder (including drug intoxication)*	1.34	1.11	1.63	0.003
**History of self-harm**				
Any psychotropic medication overdose	1.52	1.02	2.26	0.039
Hanging, strangulation or suffocation	2.66	1.43	4.93	0.002
Lifetime history of self-harm prior to index*	1.16	0.94	1.43	0.168
History of self-harm in the past 12 months prior to index, 1+ vs none*	1.36	1.07	1.73	0.012
Overnight admissions	1.76	1.46	2.11	<0.001
**Mental health in the past 12 months**				
Any psychiatric disorder except substance use disorders	1.66	1.37	2.01	<0.001
**Treatment in the past three months**				
Any psychotropic medication	2.13	1.67	2.72	<0.001
**Criminal history**				
Lifetime criminal record for violent crime prior to index	0.70	0.55	0.88	0.003
Lifetime criminal record for any crime prior to index	1.41	1.15	1.72	0.001
Exp(Intercept)	0.0014	0.0010	0.0019	<0.001
Shape parameter	0.5952	0.5477	0.6470	<0.001

*Core factor; baseline survivor function at time t, $S_0(t) = 0.0006t^{-0.4048}$.

Supplementary Table 6: Internal discrimination performance

	Apparent			Adjusted for optimism		
	Overall	6 months	12 months	Overall	6 months	12 months
	Statistic (95% CI)	Statistic (95% CI)	Statistic (95% CI)	Statistic (95% CI)	Statistic (95% CI)	Statistic (95% CI)
**Final model**						
**Harrell's c**	0.76 (0.74, 0.77)	0.77 (0.74, 0.79)	0.76 (0.75, 0.78)	0.76 (0.73, 0.78)	0.77 (0.73, 0.80)	0.76 (0.74, 0.79)
**Somers' D**	0.52 (0.48, 0.55)	0.53 (0.48, 0.58)	0.53 (0.49, 0.57)	0.51 (0.47, 0.56)	0.53 (0.47, 0.60)	0.53 (0.48, 0.58)
**Crime model**						
**Harrell's c**	0.76 (0.74, 0.78)	0.77 (0.74, 0.79)	0.77 (0.75, 0.79)	0.76 (0.74, 0.78)	0.77 (0.73, 0.80)	0.77 (0.74, 0.79)
**Somers' D**	0.52 (0.49, 0.55)	0.53 (0.49, 0.58)	0.53 (0.49, 0.57)	0.52 (0.47, 0.57)	0.53 (0.47, 0.60)	0.53 (0.48, 0.59)

Supplementary Figure 2: ROC curves in the development sample

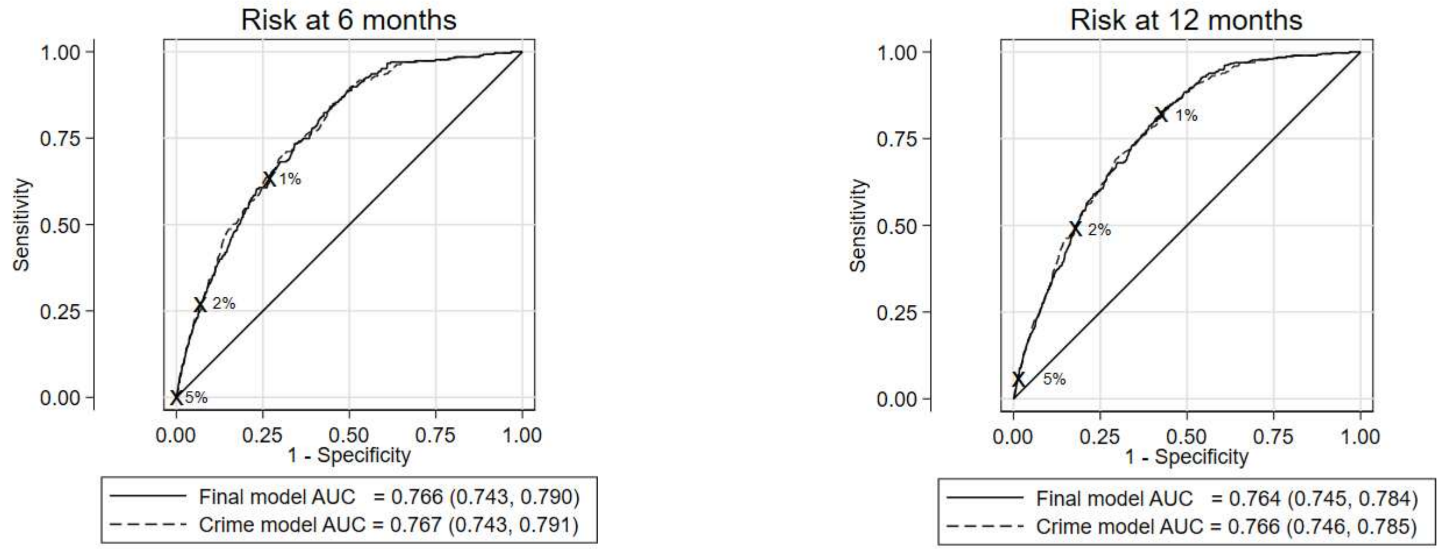

AUC = Area under the ROC curve; values are AUC and 95% confidence intervals; probability risk cut-points considered are shown (i.e. 1%, 2%, 5%).

Supplementary Figure 3: Internal calibration plots

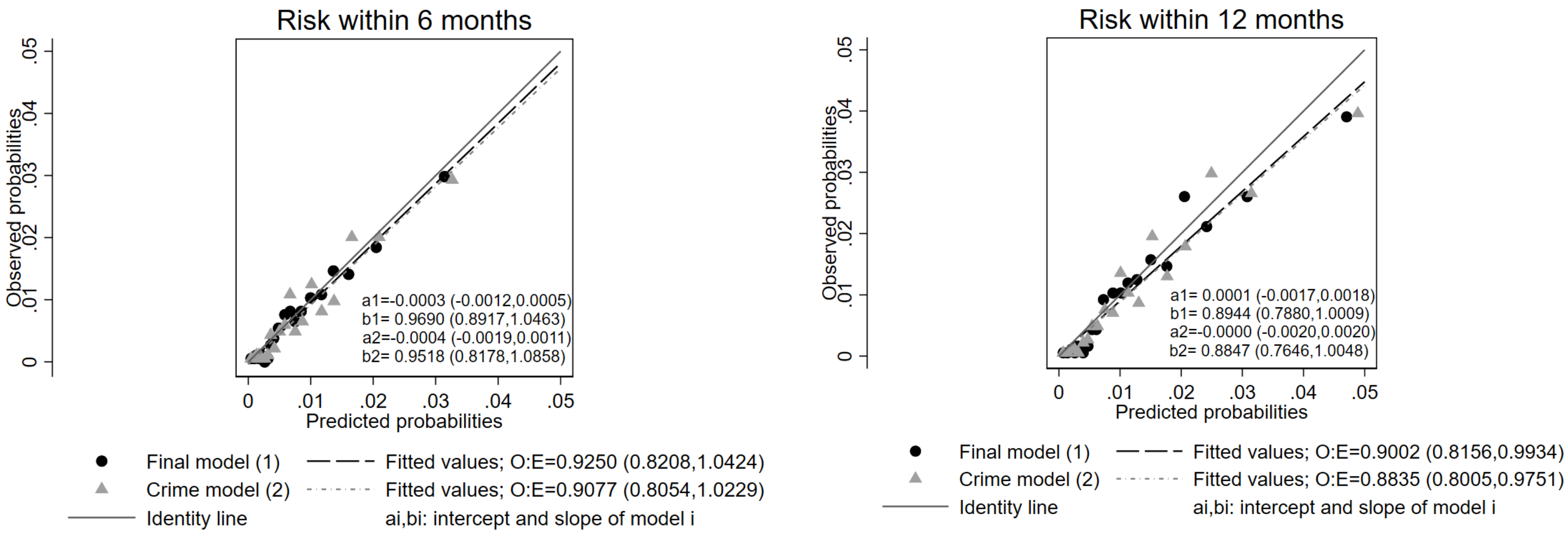

O:E = Observed to expected ratio; overall Brier score was 0.014 (95%CI 0.013 to 0.015) for both models.

Supplementary Table 7: Classification measures (and 95% confidence intervals) for the final model in the development sample

	**Risk at 6 months<br>Prevalence = 0.7% (0.6, 0.8)**			**Risk at 12 months<br>Prevalence =1.0% (0.9, 1.2)**		
**Thresholds**	**1%**	**2%**	**5%**	**1%**	**2%**	**5%**
**Sensitivity**	64.4% (58.4, 70.2)	27.7% (22.4, 33.5)	0.4% (0.01, 2.2)	82.9% (78.8, 86.5)	49.9% (44.8, 54.9)	6.7% (4.4, 9.6)
**Specificity**	71.6% (71.1, 72.0)	90.8% (90.5, 91.1)	98.1% (98.0, 98.3)	56.4% (55.9, 56.9)	80.6% (80.2, 81.0)	96.7% (96.6, 96.9)
**PPV**	1.6% (1.4, 1.9)	2.1% (1.7, 2.6)	0.1% (0.004, 0.8)	2.0% (1.8, 2.2)	2.6% (2.3, 3.0)	2.1% (1.4, 3.1)
**NPV**	99.6% (99.6, 99.7)	99.4% (99.3, 99.5)	99.3% (99.2, 99.4)	99.7% (99.6, 99.8)	99.3% (99.3, 99.4)	99.0% (98.9, 99.1)

PPV = Positive predictive value; NPV = Negative predicted value

Supplementary Table 8: Classification measures (and 95% confidence intervals) for the final model evaluated in the external validation sample

	**Risk at 6 months<br>Prevalence = 0.7% (0.6, 0.8)**			**Risk at 12 months<br>Prevalence =1.1% (1.0, 1.3)**		
**Thresholds**	**1%**	**2%**	**5%**	**1%**	**2%**	**5%**
**Sensitivity**	68% (58, 76)	24% (16, 33)	1% (0, 5)	82% (75, 87)	43% (35, 50)	7% (4, 12)
**Specificity**	71% (70, 72)	92% (92, 93)	99.9% (99.8, 99.9)	54% (53, 55)	81% (79.9, 81.1)	98.3% (98.1, 98.5)
**PPV**	2% (1, 2)	2% (1, 3)	5% (0.1, 23)	2% (1.7, 2.4)	2.5% (2, 3)	4% (2, 8)
**NPV**	99.7% (99.6, 99.8)	99.4% (99.3, 99.5)	99.3% (99.2, 99.4)	99.6% (99.4, 99.7)	99.2% (99.0, 99.3)	98.9% (98.7, 99.1)

PPV = Positive predictive value; NPV = Negative predictive value

TRIPOD Checklist: Prediction Model Development and Validation(34)

Section/Topic			Checklist Item	Page
**Title and abstract**				
Title	1	D;V	Identify the study as developing and/or validating a multivariable prediction model, the target population, and the outcome to be predicted.	1
Abstract	2	D;V	Provide a summary of objectives, study design, setting, participants, sample size, predictors, outcome, statistical analysis, results, and conclusions.	2
**Introduction**				
Background and objectives	3a	D;V	Explain the medical context (including whether diagnostic or prognostic) and rationale for developing or validating the multivariable prediction model, including references to existing models.	4
	3b	D;V	Specify the objectives, including whether the study describes the development or validation of the model or both.	4
**Methods**				
Source of data	4a	D;V	Describe the study design or source of data (e.g., randomized trial, cohort, or registry data), separately for the development and validation data sets, if applicable.	5 & 7
	4b	D;V	Specify the key study dates, including start of accrual; end of accrual; and, if applicable, end of follow-up.	5
Participants	5a	D;V	Specify key elements of the study setting (e.g., primary care, secondary care, general population) including number and location of centres.	5
	5b	D;V	Describe eligibility criteria for participants.	5
	5c	D;V	Give details of treatments received, if relevant.	NA
Outcome	6a	D;V	Clearly define the outcome that is predicted by the prediction model, including how and when assessed.	5
	6b	D;V	Report any actions to blind assessment of the outcome to be predicted.	NA
Predictors	7a	D;V	Clearly define all predictors used in developing or validating the multivariable prediction model, including how and when they were measured.	6 & Supp.Tab.1
	7b	D;V	Report any actions to blind assessment of predictors for the outcome and other predictors.	6
Sample size	8	D;V	Explain how the study size was arrived at.	6 & 8
Missing data	9	D;V	Describe how missing data were handled (e.g., complete-case analysis, single imputation, multiple imputation) with details of any imputation method.	6 & NB of Tab 1
Statistical analysis methods	10a	D	Describe how predictors were handled in the analyses.	6
	10b	D	Specify type of model, all model-building procedures (including any predictor selection), and method for internal validation.	6
	10c	V	For validation, describe how the predictions were calculated.	8
	10d	D;V	Specify all measures used to assess model performance and, if relevant, to compare multiple models.	7-8
	10e	V	Describe any model updating (e.g., recalibration) arising from the validation, if done.	NA
Risk groups	11	D;V	Provide details on how risk groups were created, if done.	7
Development vs. validation	12	V	For validation, identify any differences from the development data in setting, eligibility criteria, outcome, and predictors.	7-8

**Results**				
Participants	.3a	;V	Describe the flow of participants through the study, including the number of participants with and without the outcome and, if applicable, a summary of the follow-up time. A diagram may be helpful.	8
	.3b	;V	Describe the characteristics of the participants (basic demographics, clinical features, available predictors), including the number of participants with missing data for predictors and outcome.	10 & Tab 1
	.3c	V	For validation, show a comparison with the development data of the distribution of important variables (demographics, predictors and outcome).	10 & Tab 1
Model development	.4a	D	Specify the number of participants and outcome events in each analysis.	8
	4b	D	If done, report the unadjusted association between each candidate predictor and outcome.	Supp. Tab. 4, p.7
Model specification	.5a	D	Present the full prediction model to allow predictions for individuals (i.e., all regression coefficients, and model intercept or baseline survival at a given time point).	Supp. Tab. 3, p.6
	5b	D	Explain how to the use the prediction model.	Supp. p. 6
Model performance	16	;V	Report performance measures (with CIs) for the prediction model.	11-12 & Supp., p. 10-13
Model-updating	17	V	If done, report the results from any model updating (i.e., model specification, model performance).	NA
**Discussion**				
Limitations	18	;V	Discuss any limitations of the study (such as nonrepresentative sample, few events per predictor, missing data).	14
Interpretation	.9a	V	For validation, discuss the results with reference to performance in the development data, and any other validation data.	12-13
	9b	;V	Give an overall interpretation of the results, considering objectives, limitations, results from similar studies, and other relevant evidence.	15
Implications	20	;V	Discuss the potential clinical use of the model and implications for future research.	15
**Other information**				
Supplementary information	21	;V	Provide information about the availability of supplementary resources, such as study protocol, Web calculator, and data sets.	7-11, 14, 16
Funding	22	;V	Give the source of funding and the role of the funders for the present study.	1

*Items relevant only to the development of a prediction model are denoted by D, items relating solely to a validation of a prediction model are denoted by V, and items relating to both are denoted D;V.
